# Bonobos tend to behave optimistically after hearing laughter

**DOI:** 10.1038/s41598-025-02594-8

**Published:** 2025-06-26

**Authors:** Sasha L. Winkler, Isabelle B. Laumer, Heidi Lyn, Erica A. Cartmill

**Affiliations:** 1 Department of Anthropology, University of California, Los Angeles, CA USA; 2 Cognitive Science Program, Indiana University Bloomington, Bloomington, IN USA; 3 Development and Evolution of Cognition Research Group, Max Planck Institute of Animal Behavior, Konstanz, Germany; 4 Joan M. Sinnott Chair of Psychology, Department of Psychology and Stokes School of Marine and Environmental Sciences, University of South Alabama, Mobile, AL USA

**Keywords:** Affect, Cognition, Laughter, Ape, Optimism bias, Bonobo, Psychology, Animal behaviour, Biological anthropology

## Abstract

Emotions mediate a wide range of cognitive functions, including memory, attention, and decision making. Studies of emotion in non-human animals have typically focused on negative emotions—like fear—that have clear behavioral correlates (e.g., freezing or retreating). To address this one-sided treatment of affect, we used a cognitive bias test to ask whether vocalizations associated with positive affect lead apes to expect positive future outcomes. All great apes produce laughter-like vocalizations during play that likely evolved from a shared ancestral form of laughter. We primed bonobos with conspecific laughter and then asked whether they were more likely to treat an ambiguous stimulus as if it were positive. Subjects (*n* = 4) were first trained to approach rewarded (black) stimuli and skip unrewarded (white) stimuli. We then presented occasional ambiguous (grey) stimuli. Bonobos approached ambiguous stimuli to search for rewards more often after hearing laughter. Our results suggest that hearing laughter induces positive emotions and may thus bias bonobos’ decision making, including foraging or search behavior. While only apes produce human-like laughter, several other non-human animals have contagious play vocalizations. These vocalizations may lead other animals to anticipate positive outcomes, revealing commonalities in the role of positive emotion in behavior and cognition across species.

**Supplementary Information:**

The online version contains supplementary material available at 10.1038/s41598-025-02594-8.

## Introduction

Emotions can impact many aspects of cognition, including memory, attention, visual processing, and decision making^1,2^. Animal research has historically focused on negative emotions, but there is growing interest in positive affective states in animals^3.^ Comparative studies are well-suited to answer questions about the biological basis of affect and cognition, but are in need of robust methods for experimentally inducing positive affect^3^.

One behavior associated with positive affect in humans is laughter^4^. Despite differences in the types and contexts of human laughter, people across cultures generally recognize laughter as an expression of joy or positive emotion^5,6^. While laughter is sometimes thought of as a uniquely human trait related to humor, it has many similarities to the signals other animals make during play^7,8^. Play-specific vocalizations are common in mammals and likely evolved to reduce the risk of play being misinterpreted as aggression^8^. Great apes produce vocalizations resembling human laughter during tickling and rough-and-tumble play, much like human children^9^. Phylogenetic analyses of the acoustic properties of ape laughter suggest that these vocalizations share an evolutionary origin with human laughter^9^.

Previous research has shown that primate play vocalizations and their associated facial expression (play face) are contagious, underpinning the similarities to human laughter^10–12^. In chimpanzees, contagious laughter also correlates with longer periods of play^10^, suggesting that play vocalizations might promote positive affective states. This aligns with comparative studies showing that hearing play vocalizations induces play in kea parrots^13^, leads rats to expect positive outcomes^14^, and reduces stress behaviors in dogs^15^. Together, these studies suggest that animal play vocalizations can initiate and prolong play, and might also impact cognition outside of play.

Measuring affect in animals is difficult since one cannot use self-report. One promising approach is to look for cognitive signatures that accompany affect changes in humans^16^. We employed this method to study positive affect in bonobos (*Pan paniscus*), an ape species closely related to humans. We used a cognitive bias test to assess the emotional and cognitive effects of listening to laughter. This paradigm, alternatively called a judgement bias or ambiguous cue interpretation test^2,17^, draws on the finding that human and non-human subjects, when faced with uncertain events, expect more positive outcomes when they are in positive affective states—in other words, they are more optimistic^2^. We hypothesized that *emotional* contagion is the proximal mechanism for the *behavioral* contagion of play faces and laughter in great apes^10^, and that simply hearing laughter would induce a positive mood. We used a cognitive bias test design similar to those used in other species^14,18,19^. Subjects were first trained to produce different responses to positive and neutral stimuli. Then, we assessed their responses to ambiguous, intermediate stimuli^20^. We expected that animals in a positive mood would exhibit a greater optimism bias, and would therefore be more likely to respond to ambiguous stimuli as if they were positive.

## Results

To determine whether bonobos would respond optimistically after hearing laughter, we tested four bonobos using a within-subjects design. Subjects listened to recordings of either bonobo laughter or a control sound for exactly seven minutes and 28 s and then immediately completed a cognitive bias test that assessed their likelihood to approach ambiguous stimuli. The laughter was compiled from clips of an unfamiliar bonobo infant provided by Dr. Marina Davila-Ross. The control recording was a synthetic environmental sound (an ambient wind recording). We predicted that subjects would approach ambiguous stimuli more often after hearing laughter than they would after hearing the control sound.

**Fig. 1:**
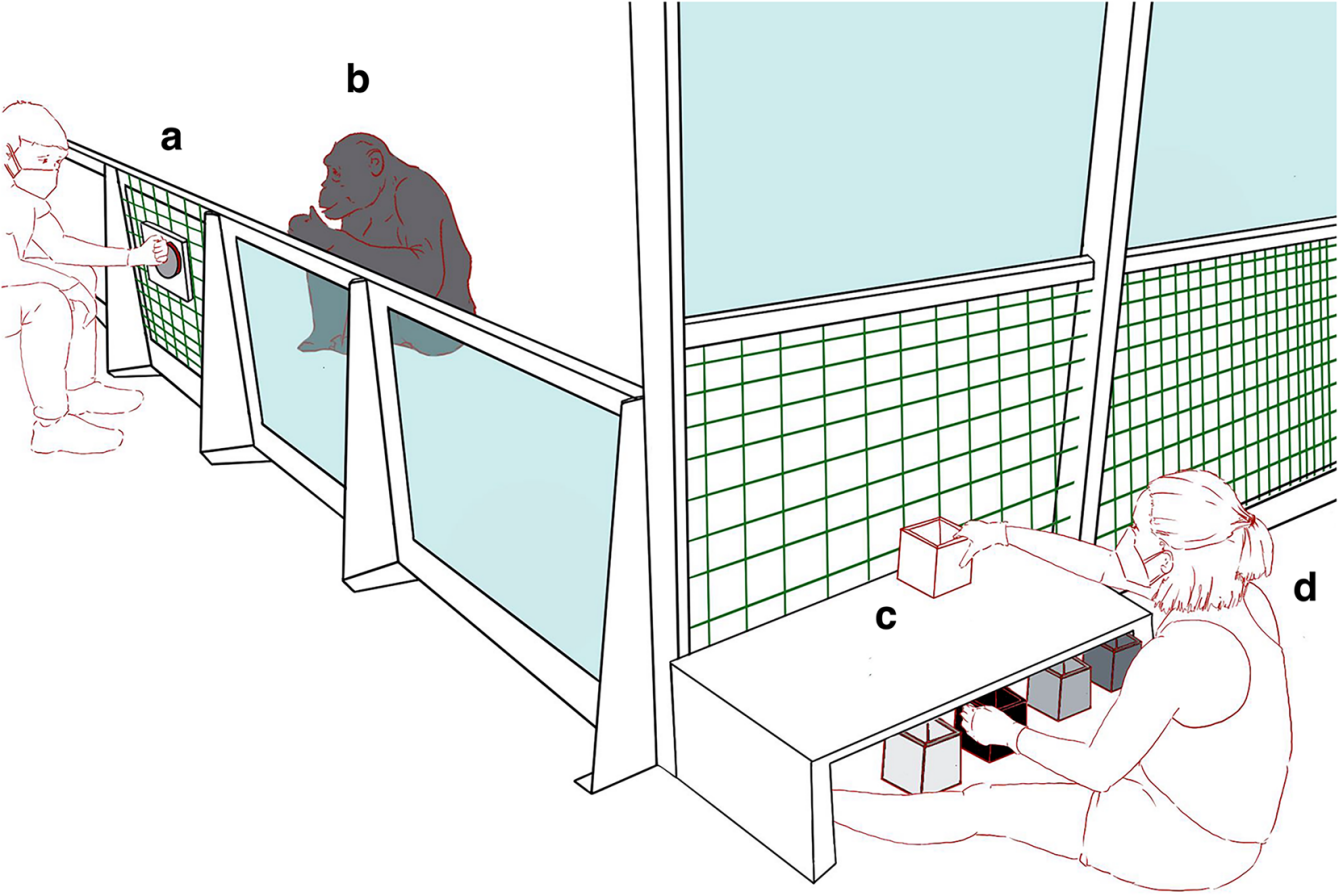
Experimental set-up. The experiment began with the bonobo (**b**) sitting close to the initiator button (**a**). Pressing the button prompted a new trial, in which the primary experimenter (**d**) presented a box (**c**) and the subject either approached (GO) or pressed the button to skip to the next trial (NO-GO). Image illustrated by Luke Townrow (CC BY).

Bonobos were first trained to approach a black box (GO) and to push an initiator button (similar to Hintze et al.^20^) to skip to the next trial when presented with a white box (NO-GO; see Fig. 1). The black (positive) box always contained a food reward, while the white (neutral) box was always empty. As in a typical judgement bias test, we assessed optimism bias—the tendency to expect positive future outcomes—as the likelihood of responding to ambiguous cues as if they signaled rewards. Thus, during testing we presented subjects with three gray (ambiguous) boxes in intermediate shades that they had never seen during training. Subjects with greater optimism bias should be more likely to approach these, expecting a reward.

**Fig. 2:**
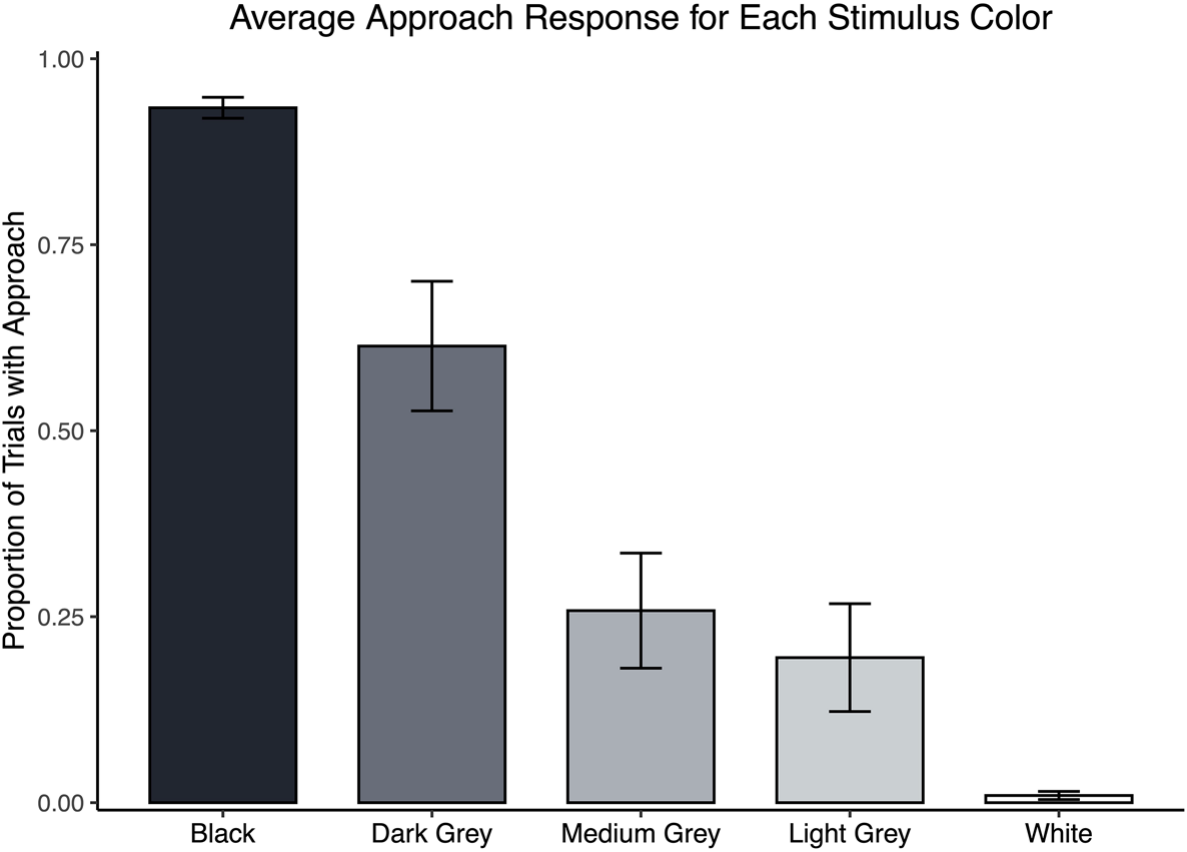
Mean proportion of testing trials with approach (GO) responses plotted against stimulus color. The black box (positive) always contained food, the white box (neutral) never did, and each grey box (ambiguous) contained food 50% of the time. Bars represent bootstrapped standard errors.

Each subject participated in four laughter and four control test sessions on separate days. Subjects were presented with ten positive, ten neutral, and three ambiguous boxes in each test session. Ambiguous boxes were rewarded 50% of the time across sessions (see Methods and SI for more details). To ensure that the paradigm worked, we calculated approach rates during testing for each of the five stimuli types. Apes almost always approached the black box (mean proportion = 0.934, *SE* = 0.014), almost never approached the white box (mean = 0.010, *SE* = 0.005), and the three intermediate stimuli were approached in accordance with their perceptual closeness to these anchor stimuli (dark grey mean = 0.613, *SE* = 0.087, medium grey mean = 0.258, *SE* = 0.077, light grey mean = 0.194, SE = 0.073, see Fig. 2). This pattern confirms that the bonobos were adequately trained on the anchor stimuli, that the color informed their approach decisions, and that the ambiguous stimuli were appropriately intermediate in color.

**Fig. 3:**
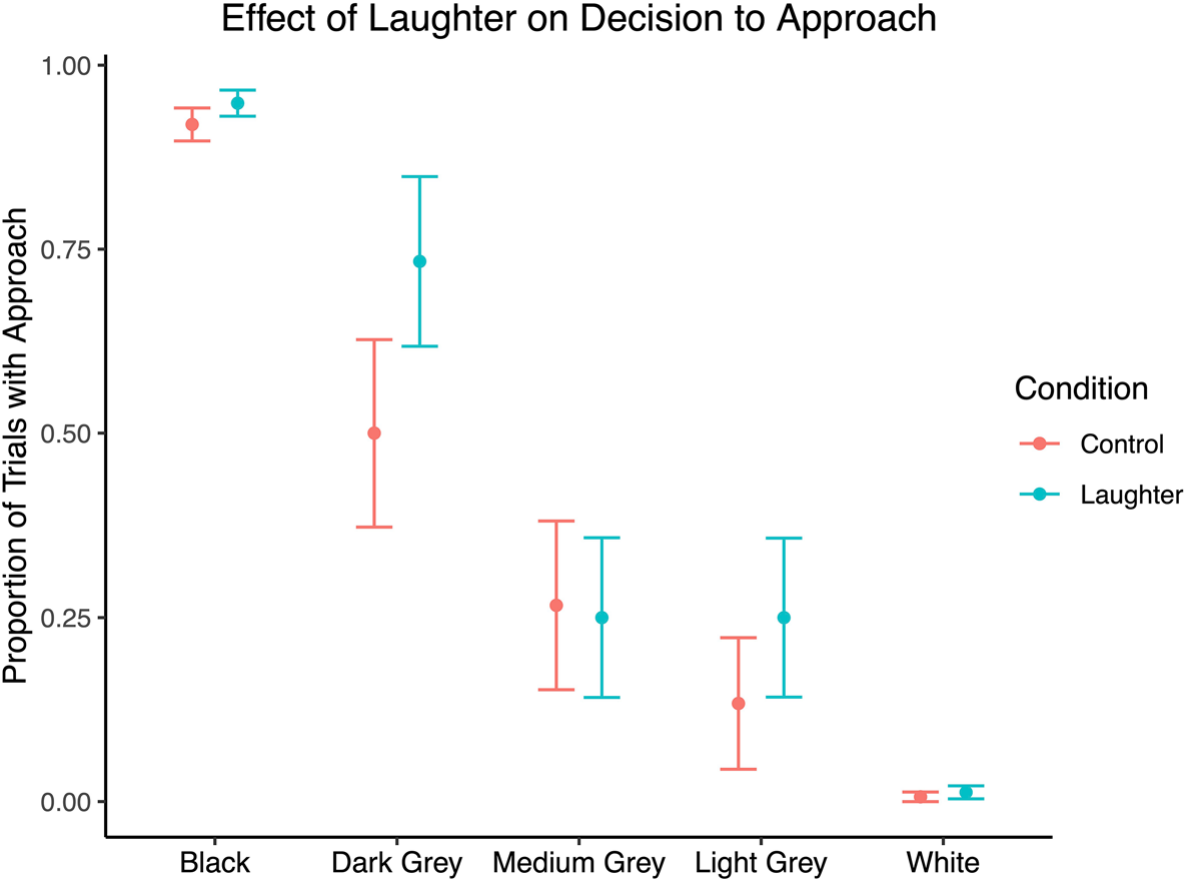
Mean proportion of testing trials with approach (GO) responses plotted against stimulus color, broken out by condition. Red = control condition (wind noise), blue = test condition (laughter). Bars represent bootstrapped standard errors.

To assess the impact of the playback condition on the decision to approach (GO) or skip to the next trial (NO-GO), we used a generalized linear regression model with binomial error distribution and logit link function. Our outcome variable was the decision to approach the ambiguous stimuli. We included condition, box color, subject, session number and date as fixed effects. After controlling for these variables, the effect of condition on the decision to approach had an odds ratio of 3.39 (95% confidence interval = 0.895 to 15.6, *p* = 0.0878). The effect of condition was marginally significant in a likelihood ratio test between the full and null model (nested model excluding condition; χ^2^ = 3.207, *p* = 0.073; see SI for additional models). Figure 3 shows the effect of condition on the likelihood of approach.

## Discussion

Bonobos tended to behave more optimistically following exposure to laughter compared to a control sound. The odds ratio for condition in our model was 3.39, meaning that bonobos in the laughter condition were expected to have more than three times greater odds of approaching the ambiguous stimuli (expecting a reward) than those in the control condition. This implies that the sound of conspecific laughter induced a positive affective state in bonobos, leading to more optimistic behavior.

To our knowledge, this study is the first to detect a positive affect shift in non-human primates from a brief experimental intervention, and the first cognitive bias test conducted with bonobos. The initial training for bonobos to reach testing criterion was quite intensive and our final sample (*N* = 4) was smaller than our target (*N* = 7) due to difficulties in training some individuals. This reduced our statistical power and was the main limitation of our study. Cognitive bias tests with response-time designs might be more practical in future research (for example, the one designed by Bateson and Nettle for use in chimpanzees^18^), as they require less training, although the animals’ expectation of reward must be inferred based on their approach times toward ambiguous stimuli, in contrast to the more easily-interpretable GO/NO-GO decision. We hope that future research will be able to expand on our study’s limitations by generalizing to larger and more diverse samples of bonobos and other great apes, and by including more varied laughter stimuli.

Our findings provide a possible emotional-communicative mechanism to explain the close coordination of motivation and behavior required of social partners during play. For example, a study of spontaneous social play in rats found that they used specific play vocalizations to coordinate complex reciprocal “moves” such as pinning and being pinned^21^. By increasing positive affect in recipients, bonobo laughter could similarly motivate reciprocation during play and help to increase positive interpretation of riskier, more ambiguous moves. Furthermore, our results highlight the importance of affect in nonverbal communication across primates by adding support for the affect-conditioning model of communication. This model proposes that communicative signals evolved to change recipients’ behavior by altering their affective states^22^.

Laughter’s positive emotional contagion appears to have been present in the primate lineage long before the evolution of language. Based on our findings, it seems likely that laughter in other apes shares not only phylogenetic and behavioral similarities with human laughter^9^ but also perhaps some of the same cognitive-emotional underpinnings. Emotional contagion, like that seen with laughter, is also thought to be an important component of empathy^23^. Further comparative studies on positive affect and its underlying cognition should build upon these findings to strengthen our understanding of emotional contagion and empathy. Ultimately, this line of research could shed light on the evolution of social bonding, prosociality, and cooperative behavior in humans and other primates.

## Methods

### Experimental model and study participant details

Subjects were four adult bonobos (three male, one female) at the Ape Cognition and Conservation Initiative in Des Moines, Iowa, USA: Mali, a 14-year-old female, Teco, a 12-year-old male, Kanzi, a 41-year-old male, and Nyota, a 24-year-old male. All animals were group-housed but voluntarily separated into solitary enclosures to participate in training and testing sessions.

All subjects had extensive experience interacting with human caretakers and participating in cognitive experiments with operant training. Three of the subjects (Kanzi, Nyota, and Teco) also had experience using lexigrams (a keyboard language system), though proficiency varied greatly between apes. Lexigrams were not used in this study, but they illustrate the level of past experience these apes had with human interaction^24^. All subjects regularly heard human laughter from their caretakers and regularly heard other bonobos in their social group laughing during play.

This study was approved by the UCLA Animal Research Committee (ARC-2022-063) and the Ape Initiative IACUC (222505-01) and was carried out in accordance with the ARRIVE guidelines and the relevant guidelines and US laws regarding animal care and use. Testing took place August to December, 2022.

### Experimental method details

Subjects listened to recordings of either bonobo laughter or a control sound and then participated in a cognitive bias test that assessed their likelihood to approach ambiguous stimuli. We applied a modified cognitive bias test design similar to one previously used in chimpanzees^18^. Stimuli were five differently-colored boxes presented approximately 3 m from the subject: one black (positive), one white (neutral), and three intermediate shades of grey (ambiguous; see Supplementary Fig. 2 to view the exact colors). After the initial training with the black and white boxes, optimism was assessed using the grey boxes.

#### Initial training

Before testing, the bonobos were trained to anticipate positive and neutral outcomes for two anchor stimuli: a positive box (black) that always contained a food reward and a neutral box (white) that never contained a food reward. Apes were also trained to use an initiator button to begin a new trial (i.e., to have a new box presented), similar to the method described in Hintze et al.^20^ If the bonobo approached to within arm’s reach of the wire mesh, the experimenter (Fig. 1d) either turned the white box on its side facing toward the bonobo, allowing them to see that it was empty, or reached into the black box and handed the grape to the bonobo, before turning it on its side in the same manner to show that the box was now empty. Over time, the bonobos learned to approach the black box (GO) and to skip to the next box when presented with a white box (NO-GO). Apes never saw the three ambiguous grey stimuli until testing.

Apes proceeded to testing if they met our training accuracy threshold. Our accuracy threshold was that a subject must approach the positive box in at least 80% of trials (GO response) and refrain from approaching the neutral box in at least 80% of trials (NO-GO response) in at least two consecutive sessions of 20 trials each (ten black and ten white, at minimum). Subjects completed an average of 37.5 training sessions of approximately 20–30 trials each before reaching accuracy (range = 23 to 55 sessions; see individual performance detailed in the SI). All seven bonobos at the Ape Initiative underwent initial training but only four reached this testing threshold. Two females were unable to reach the threshold due to low frequency of participation within the training timeframe and one male fully participated in training sessions but was unable to reach accuracy.

#### Randomization and counterbalancing

Each test subject received four laughter and four control sessions, in an alternating order counterbalanced across subjects. Test sessions consisted of 23 trials: four “refresher” trials of anchor stimuli (two positive and two neutral), followed by a semi-randomized block of eight positive, eight neutral, and three ambiguous stimuli that apes had never seen before. The ambiguous grey boxes were rewarded 50% of the time across sessions and only one or two out of three were rewarded within each session. This was designed to reduce the possibility that subjects might learn any rules or heuristics for whether to approach the ambiguous boxes over time (in contrast, if the ambiguous trials are never rewarded, animals learn to stop approaching them and likely learn faster than variable rewards). See SI for more details about the order of trials, counterbalancing, and ambiguous trial rewarding.

All sessions were videotaped, and the GO/NO-GO response was coded from video using QuickTime video player, Microsoft Excel, and BORIS software (version 7.13.8)^25^. Each subject was always tested in the same location and with the same set of experimenters. The primary experimenter interacting with the bonobo and controlling the box stimuli (Experimenter D, Fig. 1) was always blind to the experimental condition: they waited in a separate room where they could not hear the audio playback and only entered the testing room after the playback had ended. In the final analyses, we did not include any trials where the bonobo made a decision without looking at the stimuli (e.g., never looking at the box or starting to approach before the box was presented, 3.0% of trials). This ensured that all of the GO/NO-GO responses we analyzed were fully informed by the color of the box, which is key to the testing paradigm. We also excluded trials where the experimenter made mistakes with the stimuli presentation (e.g., incorrect timing, 0.5% of trials; see SI). Our first attempted test session with Nyota was excluded from analysis as he refused to continue participating halfway through the cognitive test. After a delay of 18 days, we restarted testing with Nyota and added six additional anchor stimuli trials before each playback to allow experimenters to assess his motivation to participate in research that day (see SI). No other sessions experienced significant interruptions.

#### Audio playback information

The laughter playback recording was compiled from clips of an unfamiliar infant bonobo laughing while being tickled by a human experimenter. The clips were provided by Dr. Marina Davila-Ross at the University of Portsmouth. The laughs were from the same bonobo, a male approximately eight months old. Several shorter laughter clips were edited and integrated into one 3:44 clip that was repeated with 1 s. of silence in between. The total duration of the playback recording was 7:28. A small amount of mechanical, percussive, and/or ambient noise was present in some of the clips used to produce the playback recording. All audible human sounds were edited out. In cases where we could not remove the human-generated sounds without affecting the bonobo laughter sounds, we did not include the entire clip in constructing the playback.

The control recording was a time- and volume- matched recording of a sound similar to white noise. Instead of using true white noise, which can be aversive to some animals^14,26^, we used a synthetic environmental wind noise similar to those available on commercial sleep machines (a “winter storm” mp3 purchased on Soundcloud). The duration was the same as the final laughter audio (7:28, including a short fade in and out to avoid a harsh onset/offset) and the average root-mean-square amplitude was matched using Adobe Audition. Audio was played from an Anchor AN-MINI speaker connected to a laptop, which was set to a consistent volume level across all sessions. The audio was controlled using either Windows Media Player or Apple Music software.

Before the playback began, subjects voluntarily entered the testing enclosure alone, with no visual or tactile access to other bonobos for the duration of testing. All subjects listened to the audio stimuli for exactly seven minutes and 28 s. During this time, subjects were free to move around the enclosure. One experimenter (Experimenter A, Fig. 1) was present to operate the laptop and video camera—this experimenter stayed at least three feet away from the enclosure, averted their gaze, and did not interact with the bonobo during the playback. The blind experimenter (the primary experimenter operating the black, white, and grey boxes, Experimenter D, Fig. 1) waited in a different room, separated by at least two doors, with no ability to hear the playback. After the audio clip ended, the blind experimenter was immediately notified via radio, entered the room, and began the cognitive bias trials. Each test session lasted approximately 15 min (mean = 15.3, range = 12.3 to 19.3 min), including the playback stimulus, all of the test trials, and a few minutes for the blind experimenter to enter the room and prepare the stimuli.

We were limited to one control condition due to the small sample size and experimental design. Having fewer conditions allowed us to keep the number of testing sessions low, and thus limit the total number of ambiguous trials seen by each subject. This lowered the risk that bonobos would learn rules or heuristics for responding to the ambiguous stimuli. We opted to play a neutral environmental sound as the control condition to keep all the experimenters’ behavior between the conditions as similar as possible (setting up the speaker, playing sounds from a laptop, etc.) between conditions. While a neutral vocalization would be an ideal control, we could not identify any bonobo vocalization that would be predicted to be meaningless or without affective relevance to the listener. Future research with larger sample sizes would benefit from additional controls to assess the effects of laughter compared to other vocalizations.

### Quantification and statistical analysis

All statistical models were fitted in R version 4.3.1^27^. Our primary statistical model assessing the effect of condition on the likelihood of approaching was fit using the glm function in the stats package^28,29^ using the binomial family and the following model formula: *Approach* ~ *Condition* + *Stimulus Color* + *Date* + *Testing Session* + *Bonobo*. *Approach* was the decision to GO (coded as 1) or NO-GO (coded as 0). *Condition* was laughter (1) or control (0). *Stimulus Color* was a categorical variable for the color of the box (Dark Grey, Medium Grey, or Light Grey). *Date* was the day of testing, with 0 being the first day of testing for any bonobo, included to control for exogenous mood effects related to seasonality as testing began in summer and ended in winter. *Testing Session* was the session number as a categorical variable included to control for order effects. *Bonobo* was the subject’s identifier, included as a fixed effect.

We conducted a likelihood ratio (Chi-squared) test to estimate the effect of including *Condition* in the model. We compared the goodness of fit between two nested models, the full model including *Condition* and a simpler model including the same predictors except for *Condition*, to evaluate whether the full model improved the fit to the data compared to the simpler model^30^. See the SI and code for more modeling details.

The standard errors for Figs. 2 and 3 were calculated using a bootstrap method using the function boot() in the boot package in R^31,32^. The original data was resampled with replacement to produce a distribution of 2000 bootstrapped sample means.

The data and code are publicly available on the Open Science Foundation website: https://osf.io/jpn9a/.

## Electronic supplementary material

Below is the link to the electronic supplementary material.

Supplementary Material 1.

## Data Availability

The data and code are publicly available on the Open Science Foundation website: https://osf.io/jpn9a/.
