# Supplementary material for: Bonobos tend to behave optimistically after hearing laughter
Source: Sci Rep. 2025 Jun 26;15:20067. doi: 10.1038/s41598-025-02594-8 (PMC12202794; doi:10.1038/s41598-025-02594-8)
Supplement: Supplementary file 1 — Supplementary Material 1. [file 41598_2025_2594_MOESM1_ESM.pdf]

Supporting Information for

Bonobos tend to behave optimistically after hearing laughter

Authors: Sasha L. Winkler*[1,2], Isabelle B. Laumer[3], Heidi Lyn[4], Erica A. Cartmill*[1,2]

Affiliations: [1]Department of Anthropology, University of California, Los Angeles, California, USA; [2]Cognitive Science Program, Indiana University Bloomington, Bloomington, Indiana, USA; [3]Development and Evolution of Cognition Research Group, Max Planck Institute of Animal Behavior, Konstanz, Germany; [4]Joan M. Sinnott Chair of Psychology, Department of Psychology and Stokes School of Marine and Environmental Sciences, University of South Alabama, Mobile, Alabama, USA

*Corresponding authors: Sasha L. Winkler, Erica A. Cartmill
Author contact: sashawinkler@g.ucla.edu, ericac@iu.edu

This PDF file includes:

Supporting text
Supplementary Table 1
Supplementary Table 2
Supplementary Table 3
Supplementary Figure 1
Supplementary Figure 2
SI References

Other supporting materials for this manuscript include the following:

Dataset S1 (https://osf.io/jpn9a/)
Software S1 (https://osf.io/jpn9a/)

Supporting Text

Additional training details
After the initial training stage with just the initiator button, the training sessions were typically 20-30 trials, although we occasionally did 40 or more trials if the bonobo was highly engaged. A 20-30 trial training session lasted approximately 10 minutes, with the speed typically increasing as training progressed.

Supplementary Table 1. Description of training stages

Training stage	Number of trials	Distance between initiator and rewards	Description of trials
(1) Initiator button only	8-31	Within arm's reach (<3 feet)	100% rewarded for pressing button, no boxes
(2) Initiator button + 100% reward rate + close distance	20+	Within arm's reach (<3 feet)	100% rewarded, black boxes presented only
(3) Initiator button + variable reward rate + increasing distance	20+	Progressively increasing distance between 3-10 feet based on each bonobo's speed of learning	Mix of black and white boxes, 90-60% black rewarded trials progressing toward 50% based on each bonobo's speed of learning
(4) Final training stage	20+	Initiator located approximately 10 feet from the rewards (identical to testing conditions)	50% rewarded, half black and half white boxes

Supplementary Table 2. Description of training sessions for each subject.

	Bonobo name			
	**Mali**	**Teco**	**Kanzi**	**Nyota**
Total # of training sessions before testing	23	34	37	55
Sessions with 100% rewards (stages 1 and 2)	7	7	7	7
Sessions with variable rewards and increasing button distance (stage 3)	8	20	17	33
Sessions in final training stage (stage 4)	8	7	13	15
Accuracy on black boxes, final session #1	100%	100%	80%	100%
Accuracy on white boxes, final session #1	86.7%	100%	100%	90%
Accuracy on black boxes, final session #2	100%	100%	80%	91.7%
Accuracy on white boxes, final session #2	91.7%	100%	93.3%	100%

Supplementary Table 3. Description of training sessions for bonobos who did not reach criteria for testing.

	Bonobo name		
	**Elikya**	**Clara**	**Maisha**
Total # of training sessions	20	27	30
Sessions with 100% rewards (stages 1 and 2)	10	6	7
Sessions with variable rewards and increasing button distance (stage 3)	10	21	23
Sessions in final training stage (stage 4)	0	0	0
Accuracy on black boxes, final session	94.4%	100%	30%
Accuracy on white boxes, final session	25%	69.2%	100%
Final training stage achieved	60% rewards, ~0-3 ft distance	50% rewards, ~4-5 ft distance	60% rewards, ~4-5 ft distance
Training issues	Poor accuracy with increasing distance, low motivation to separate, low motivation to finish sessions, difficulty learning to skip trials	Poor accuracy with increasing distance, low motivation to separate	Poor accuracy, difficulty inhibiting button press

Additional audio information

The playback speaker was placed 3-5 feet away from the enclosure in the same location for each testing room, and each bonobo was tested in the same room for each of their test sessions. The unblinded experimenter sat near the speaker at the beginning and end of the playback and operated a handheld video camera. The experimenter moved around as needed to maintain the subject in view of the camera, but averted their gaze and did not interact with the bonobo in any way.

The playback length of 7 min 28 seconds was chosen because we wanted the laughter to persist long enough to induce a persistent affective state or "mood" in the listener. A previous study in rats[S1] played positive or negative vocalizations to rats intermittently for 20 minutes, which was found to successfully induce an emotional state as detected by a cognitive judgement bias test. We chose to repeat the concatenated laughter audio twice as a tradeoff between having sufficient time to induce a persistent affective state and avoiding too much repetition, which would risk boredom or habituation.

We used laughter from a young animal so that the study could be replicated in other apes such as chimpanzees, who may respond negatively to unknown adult vocalizations since outgroup adults present lethal threats in the wild.[S2,S3] Spontaneous laughter recorded during conspecific

bonobo play typically includes overlapping vocalizations from two or more individuals, while tickling by a human experimenter produces cleaner audio files with just one vocalizer. We did not have the ability to record tickling with all individuals at the Ape Initiative, therefore we used prerecorded laughter of tickling sessions with an unknown young male provided by Dr. Marina Davila-Ross to construct the longer audio. Future research should explore the possibility that bonobos can recognize laughter from specific individuals and use larger samples to explore the ways that factors such as familiarity, sex, age, dominance, or relationship quality could impact the perception of conspecific laughter.

Order of testing sessions
All bonobos received laughter and control sessions on separate days in an alternating order, with two subjects starting with laughter and two subjects starting with control. However, due to an experimenter error, one bonobo (Mali) received the order (1) control, (2) laughter, (3) control, (4) laughter, (5) control, (6) control, (7) laughter, (8) laughter—the order of sessions 6 and 7 was swapped. All other sessions were counterbalanced, and we controlled for condition and session number in the statistical models.

Order of trials within sessions
Each trial involved a single stimulus presentation (including the bonobo pressing the initiator button, the experimenter presenting a new box, and the bonobo's GO/NO-GO decision). Each testing session consisted of 23 trials total: ten positive/black trials, ten neutral/white trials, and three ambiguous/grey trials (each one a different shade of grey).

For randomization purposes, we split the 23 trials into two blocks, although these were presented seamlessly to subjects without any breaks. The first randomized block was two black boxes and two white boxes (four trials total), pseudorandomized such that we always started with a black. These were referred to as the "refresher" trials. The refresher trials always came first and were designed to increase motivation at the start of the session and to refresh the bonobo's anchor stimuli training.

One subject (Nyota) exhibited low motivation to participate in any research task on certain days, leading him to often have incomplete training sessions and an incomplete attempt at his first testing session (see "Excluded trials" below). To avoid further issues during testing, we assessed Nyota's willingness to participate *before* we started the audio playback, by presenting him with an additional refresher block of 6 trials (3 black, 3 white, pseudorandomized to always start with a black box) before testing started. If he refused to engage in these 6 probe trials, testing was postponed to the next available testing day.

After the refreshers (10 trials total for Nyota, 4 for the others), the apes were presented with the remaining 19 trials: eight black, eight white, one light grey, one medium grey, and one dark grey. These were pseudorandomized such that the ambiguous grey trials were not clustered together and no more than three trials of one type of anchor (or one type of anchor stimuli plus ambiguous stimuli) appeared in a row. This was to prevent learning of any heuristic responses and to sustain participation, because bonobos tended to lose motivation if too many unrewarded trials appeared in a row during training.

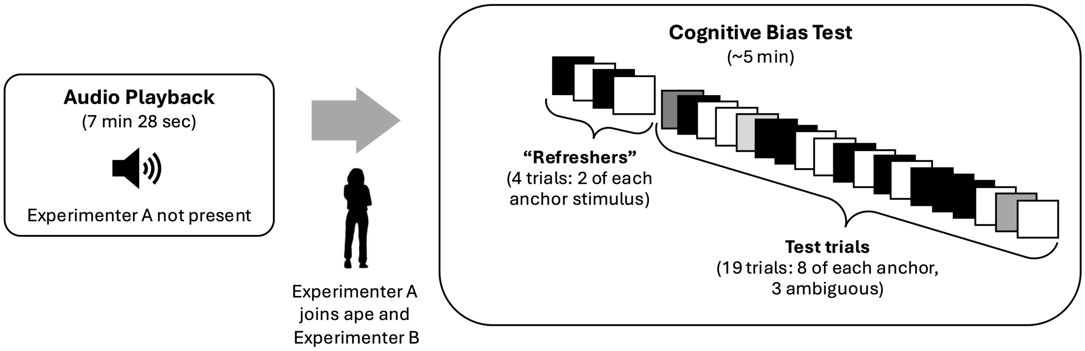

Supplementary Figure 1. A visualization of the standard testing protocol. Each testing session consisted of an audio playback phase, during which the primary experimenter A was not present, followed by a cognitive bias test. The cognitive bias test consisted of 23 trials, with the first 4 considered refresher trials and the remaining trials pseudorandomized according to the rules described in the supplementary text. Testing sessions lasted approximately 15 minutes total including the audio phase, the cognitive bias test, and time for the experimenter to enter the room and set up the test. For one subject (Nyota), we introduced an additional motivational probe prior to the standard testing protocol, which consisted of 6 anchor trials, to assess his willingness to participate in research that day.

Ambiguous trial rewarding

We randomized rewards for the ambiguous stimuli (the three grey boxes). Randomization was designed to reduce the possibility that subjects might learn any rules or heuristics for whether to approach the ambiguous boxes. Grey boxes were rewarded on average 50% across all the trials that each bonobo received. These were pseudorandomized such that the three ambiguous trials within a testing block were never all unrewarded or all rewarded. The reward rate was (1) 50% for *all* shades of grey in total across all sessions; (2) 50% for *each* shade of grey across all sessions; (3) 50% for *all* shades of grey in the laughter sessions and 50% in the control sessions; (4) either 33% or 66% across *all* the shades of grey *within* each session (since there were only three grey trials per session). Due to an experimenter error, there was one ambiguous trial that was unrewarded when it should have been rewarded (subject Mali, session 7, dark grey trial) resulting in 33% ambiguous trials rewarded in that session rather than 66%.

Excluded trials

In the final analyses, we did not include any trials where the bonobo or experimenters made mistakes with the testing paradigm (3.5% of trials). Mistakes were mostly trials in which the bonobos did not see the box color before making a decision: either the bonobo started to approach before the box was visible and the experimenter, not noticing this, still presented a box (i.e., false start; 1.8%), or the bonobo did not turn their head to look toward the box at any point before pressing the button for the next trial (1.2%). This ensured that all of the GO/NO-GO responses we analyzed were fully informed by the color of the box, which is key to the testing paradigm. Four trials were excluded due to human experimenter errors with the testing procedure (0.5%; two accidentally skipped trials and two trials with mistakes on the timing of the button replacement). One additional anchor trial was mistakenly added at the end of a session (subject Teco, session 1, black trial) and was included in analyses of the anchor stimuli.

Our protocol if a bonobo walked away or became distracted during testing was that the primary experimenter was instructed to silently count to 10 and then say “try again,” “do you want to keep going?,” or “let’s keep going” in a neutral voice to encourage the subject to begin the next trial (i.e., to press the initiator button). The bonobos in our study were used to hearing spoken English and being prompted by experimenters, so while they may not have understood the sentences, the intent should have been familiar to them. The experimenter could repeat this process of silently counting to 10 and prompting up to 3 times, and if the subject did not successfully re-engage in the study after 3 attempts, the experimenter aborted the session.

In all but one of the testing sessions, the bonobos proceeded from the audio playback directly into the trials and completed the test sessions without any interruptions. The one exception was during our first attempt at testing Nyota, during which he walked away midway through the cognitive bias test and refused to participate even after 3 attempts to reengage him. We aborted this session, and waited 18 days to restart testing to reduce any possible habituation effects (with several training sessions during the delay before testing restarted). This meant that Nyota had one exposure to the laughter audio and one exposure to a single light grey box before the four full testing sessions began. Other than this, we did not need to abort or exclude any other test sessions in our study. We were able to avoid any other extra exposures to the audio stimuli, as every other audio playback we started was immediately followed by a complete testing session. There were also no other significant interruptions: other than the one aborted session with Nyota, which was not included in the final data, the longest time between the start of two consecutive trials was approximately 40 seconds, including the normal time to approach the box, receive the reward, and return to the initiator button.

Random effect model

As we had no subject-level research questions we included *Bonobo* as a fixed effect. This is standard practice when there are fewer than five levels of the cluster variable (see [S4] for a nuanced discussion of these modeling tradeoffs). A multilevel version of the same model with *Bonobo* as a random intercepts effect was fit using the glmer function in the lme4 package[S5] using the binomial family with the optimizer “bobyqa” to aid in convergence. We used the following model formula: *Approach ~ Condition* + *Stimulus Color* + *Date* + *Testing Session* + (1 | *Bonobo*). After accounting for the control variables, the effect of condition in this model had an odds ratio of 2.79 (95% confidence interval = 0.795 to 9.77, p = 0.109). The likelihood ratio test between the full and nested model excluding condition resulted in a χ^2 of 2.7051 (p = 0.100).

Supporting information for Figure 2

In Figure 2, the mean proportion of trials with approach for the **black box** across all testing conditions = 0.934, *SD* = 0.248, *SE* = 0.014, number of trials = 304, 95% *CI* = [0.905, 0.961]. The mean approach proportion for the **dark grey box** across all testing conditions = 0.613, *SD* = 0.495, *SE* = 0.087, number of trials = 31, 95% *CI* = [0.452, 0.774]. The mean approach proportion for the **medium grey box** across all testing conditions = 0.258, *SD* = 0.445, *SE* = 0.077, number of trials = 31, 95% *CI* = [0.129, 0.419]. The mean approach proportion for the **light grey box** across all testing conditions = 0.194, *SD* = 0.402, SE = 0.073, number of trials = 31, 95% *CI* = [0.065, 0.355]. The mean approach proportion for the **white box** across all testing conditions = 0.010, *SD* = 0.097, *SE* = 0.005, number of trials = 314, 95% *CI* = [0, 0.0223].

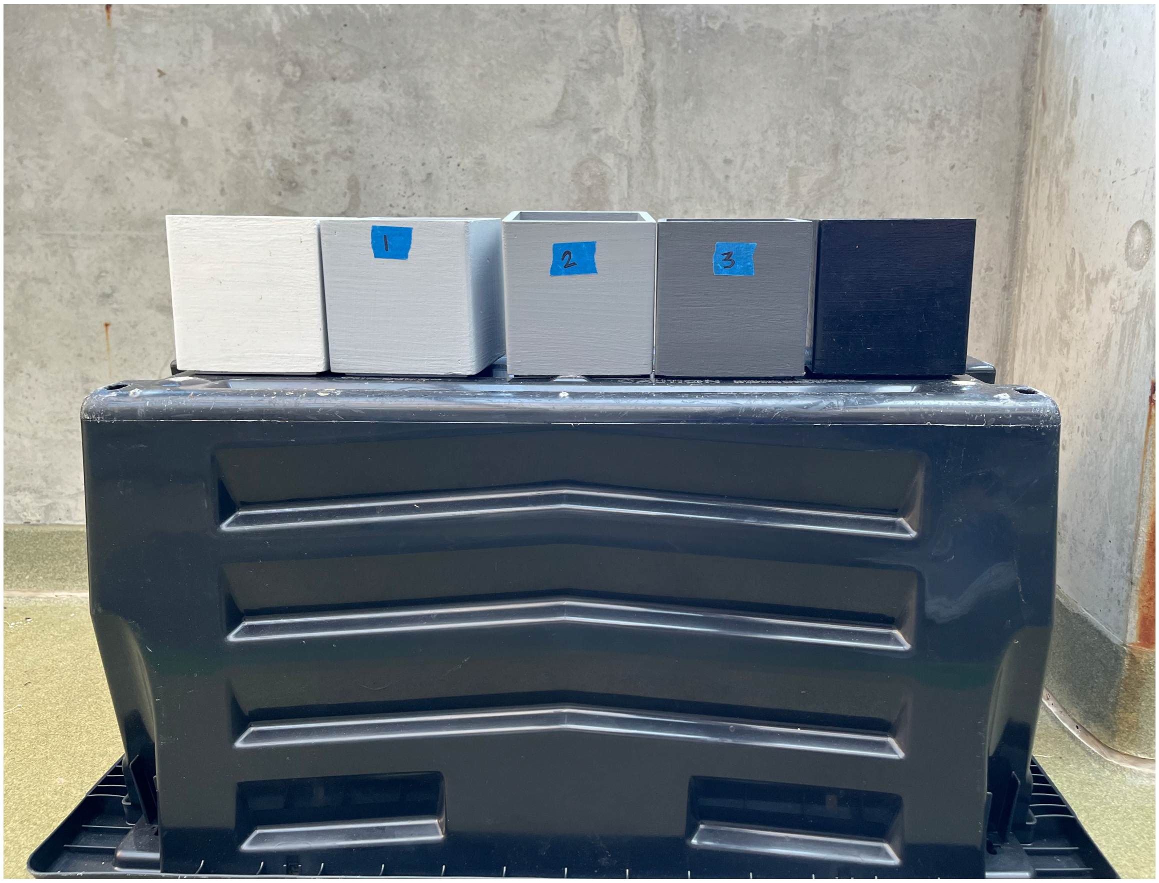

Supplementary Figure 2. A photograph of the five box stimuli. The box stimuli are pictured on the table used in the study; from left: white, light grey, medium grey, dark grey, and black. Note that the blue stickers pictured here were only used by experimenters and were never visible to the bonobos.

SI References

S1. Saito, Y., Yuki, S., Seki, Y., Kagawa, H., and Okanoya, K. (2016). Cognitive bias in rats evoked by ultrasonic vocalizations suggests emotional contagion. Behavioural Processes *132*, 5–11. https://doi.org/10.1016/j.beproc.2016.08.005.

S2. Baker, K.C., and Aureli, F. (1996). The neighbor effect: Other groups influence intragroup agonistic behavior in captive chimpanzees. American Journal of Primatology *40*, 283–291. https://doi.org/10.1002/(SICI)1098-2345(1996)40:3<283::AID-AJP5>3.0.CO;2-U.

S3. Campbell, M.W., and De Waal, F.B.M. (2011). Ingroup-Outgroup Bias in Contagious Yawning by Chimpanzees Supports Link to Empathy. PLOS ONE *6*, e18283. https://doi.org/10.1371/journal.pone.0018283.

S4. Gomes, D.G.E. (2022). Should I use fixed effects or random effects when I have fewer than five levels of a grouping factor in a mixed-effects model? PeerJ *10*, e12794. https://doi.org/10.7717/peerj.12794.

S5. Bates, D., Mächler, M., Bolker, B., and Walker, S. (2015). Fitting linear mixed-effects models using lme4. Journal of Statistical Software *67*, 1–48. https://doi.org/10.18637/jss.v067.i01.
